# Dilution and the theoretical description of growth-rate dependent gene expression

**DOI:** 10.1186/1754-1611-7-22

**Published:** 2013-09-16

**Authors:** Marius Hintsche, Stefan Klumpp

**Affiliations:** 1 Max Planck Institute of Colloids and Interfaces, Science Park Golm, 14424, Potsdam, Germany

**Keywords:** Genetic circuits, Modeling, Bacterial growth, Dilution, Growth-rate dependence

## Abstract

Expression of a gene is not only tuned by direct regulation, but also affected by the global physiological state of the (host) cell. This global dependence complicates the quantitative understanding of gene regulation and the design of synthetic gene circuits. In bacteria these global effects can often be described as a dependence on the growth rate. Here we discuss how growth-rate dependence can be incorporated in mathematical models of gene expression by comparing data for unregulated genes with the predictions of different theoretical descriptions of growth-rate dependence. We argue that a realistic description of growth effects requires a growth-rate dependent protein synthesis rate in addition to dilution by growth.

## 

Genetic circuits are unavoidably coupled to the global state of their host cell, which provides the machinery for gene expression [1]. Rather than being a rigid “chassis” for these gene circuits, the host cell itself is dynamic and adapts to external conditions, complicating the predictive design of synthetic gene circuits [2,3]. In bacterial cells, the most important characteristic of the global state of the cell is the growth rate, and pmeters of the host cell (such as the ribosome and RNA polymerase content) can often be described as growth-rate dependent [4]. Aiming at a quantitative understanding of genetic circuits, a number of recent studies have therefore addressed growth-rate dependent effects [1,5-9]. In experiments, such global effects can be studied by comparing the expression of regulated genes with an unregulated reference gene that only reflects global changes in gene expression. To include the global effects in mathematical models of gene circuits, a mathematical description of the growth-rate dependence of an unregulated reference gene is required. In this letter we discuss and compare several version of such mathematical description and compare their predictions with a compilation of experimental data [1].

In many modeling approaches, the dynamics of the concentration (*p*) of a protein is described by a balance of synthesis and (effective) degradation with rates α and β, respectively,

(1) $$\frac{d}{\mathrm{dt}}p=a-\mathrm{\beta p}$$

The synthesis rate α includes the rates of transcription and translation as well as the degradation rate of the corresponding mRNA and the concentration of the corresponding gene. For regulated genes, α is furthermore a function of the concentrations of the corresponding transcription factors or other regulators. Here, to septe global growth effects and specific regulation, we consider unregulated (constitutive) gene expression, without such dependencies. The degradation rate in this equation is an effective degradation rate, β=β_0_+λ, given by the sum of the rates of actual degradation by proteolysis (β_0_) and of dilution due to cell growth with the dilution rate or growth rate λ (which is related to the doubling rate μ via λ=μ ln2). Most proteins in *E. coli* are not actively degraded during balanced growth [10], so typically dilution by cell growth is dominant, β≈λ.

When effects of growth are considered in models of gene circuits, they are often identified with this dilution effect and the synthesis rate α is assumed to be constant, independent of the growth rate. As a result, the steady state protein concentration is proportional to the inverse of the growth rate, *p*(λ)=α/λ. Thus, if these assumptions are correct, the product of growth rate and the concentration of a constitutively expressed protein, *p*(λ)×λ, should be constant (black lines in Figure 1). In Figure 1 we plot this product (which corresponds to an effective synthesis rate) using a compilation of data from ref. [1] for the growth rate dependence of protein concentrations for unregulated genes in *E. coli*. Despite the scatter in the data at the highest growth rates, the product can be seen to be approximately constant for moderate to rapid growth, but it clearly decreases when the growth rate is reduced below 1 doubling/hour.

**Figure 1:**
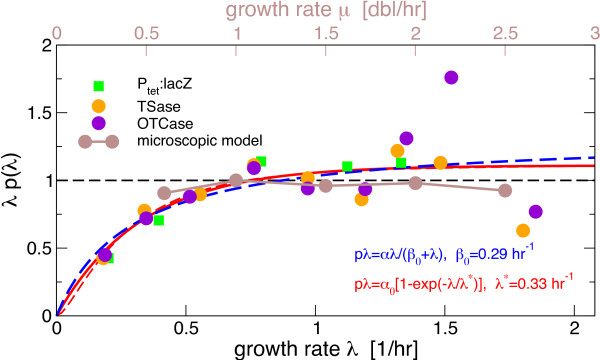
**Growth-rate dependence of the product *p*×λ of the concentration of an unregulated protein (*p*) and the growth rate (λ).** For stable proteins, this product corresponds to the protein synthesis rate. The lines show the predictions from models with dilution only (dashed black), dilution plus growth rate independent degradation (blue) and dilution plus growth rate dependent synthesis (red), the latter two are fitted to the data with the indicated pmeters. The dashed red line modifies the last case by also including a small degradation rate. The data is a compilation of growth-dependent concentration of several unregulated proteins from ref. [1]. The concentrations are normalized to their value at 1 doubling/hour.

This additional growth dependence could be explained by a growth dependent synthesis rate or be due to a different growth dependence of the degradation rate. The next simplest possibility is to include active degradation with a constant degradation rate β_0_. Then the steady state of a constitutive gene reads *p*(λ)=α/(β_0_+λ) and the product *p*×λ is given by αλ/(β_0_+λ). A least square fit of this hyperbolic function to the data leads to the blue curve in Figure 1 with a degradation rate β_0_ = 0.29 hr^−1^, corresponding to a protein half-life of ~2.4 hours. This value is much smaller than the half-lifes measured for the bulk of proteins in exponentially growing *E. coli*, which has been determined to be ≥10 hours [10,11]. Likewise, if we specifically fit the data with a beta-galactosidase reporter (P_tet_:lacZ data from ref. [1]), we obtain a half-life of ~1.4 hours, also much smaller than the value of ≥10 hours reported for this protein [12]. We therefore conclude that this approach to modeling growth-rate dependence, while providing a good fit to the data, overestimates protein degradation.

Thus, the growth-rate dependence of p×λ has to be attributed either to a growth-rate dependent protein synthesis rate α or to a growth-rate dependent degradation rate β_0_. While the latter case cannot be excluded, the extent of growth-rate dependent effects in protein degradation is not very clear. Increased degradation rates have been reported for non-growing or slowly growing cells, but either with small degradation rates (≈0.05 hr^-1^) [13] or with only a small fraction of protein (2–5%) that is rapidly degraded [10]^a^. On the other hand, a decrease of the protein synthesis rate at slow growth has recently been reported [9], likely reflecting the reduced availability of RNA polymerases under these conditions [14]^b^. Assuming again stable proteins, i.e. β=λ, an excellent fit to the data in Figure 1 is obtained with the exponential dependence

(2) $$a\left(\lambda \right)=a_{0}\left[1-\exp\left(-\lambda /\lambda^{*}\right)\right]$$

The resulting curve with λ^*^ = 0.33 hr^-1^ is shown in red in Figure 1. We note that protein degradation cannot be neglected for arbitrarily slow growth, but including a degradation rate β_0_ by fitting with pλ=α(λ)×λ/(λ+β_0_) has only a minor effect on the fit (dashed red line in Figure 1, with λ^*^ = 0.24 hr^-1^,β_0_ = 0.06 hr^-1^).

In summary, we propose to describe an unregulated reference gene by dilution and a growth rate dependent protein synthesis rate (Equation 2). The two simplest and often-used descriptions of growth effects are not sufficient: A description with constant synthesis and degradation rates and dilution is only consistent with the data if an unrealistically high degradation rate is assumed. The data is clearly inconsistent with models where growth only affects the dilution of stable proteins.

## Endnotes

^a^For comparison, attributing all growth rate dependence of *p*(λ)×λ to the degradation rate, β_0_ would be given by λ^*^ from Equation 2 at slow growth.

^b^The reported synthesis rates are for a constant gene concentration. This condition is expected to be approximately fulfilled for chromosomal genes at slow growth, where the gene copy number is given by the copy number on the chromosome and the cell volume varies only weakly [4].

## Competing interests

The authors declare that they have no competing interest.

## Authors’ contributions

MH and SK conceived and performed the analysis. SK wrote the paper. Both authors read and approved the final manuscript.
